# Supplementary figures and images for: Round goby (Neogobius melanostomus) δ13C/δ15N discrimination values and comparisons of diets from gut content and stable isotopes in Oneida Lake
Source: PLoS One. 2023 Apr 24;18(4):e0284933. doi: 10.1371/journal.pone.0284933 (PMC10124857; doi:10.1371/journal.pone.0284933)

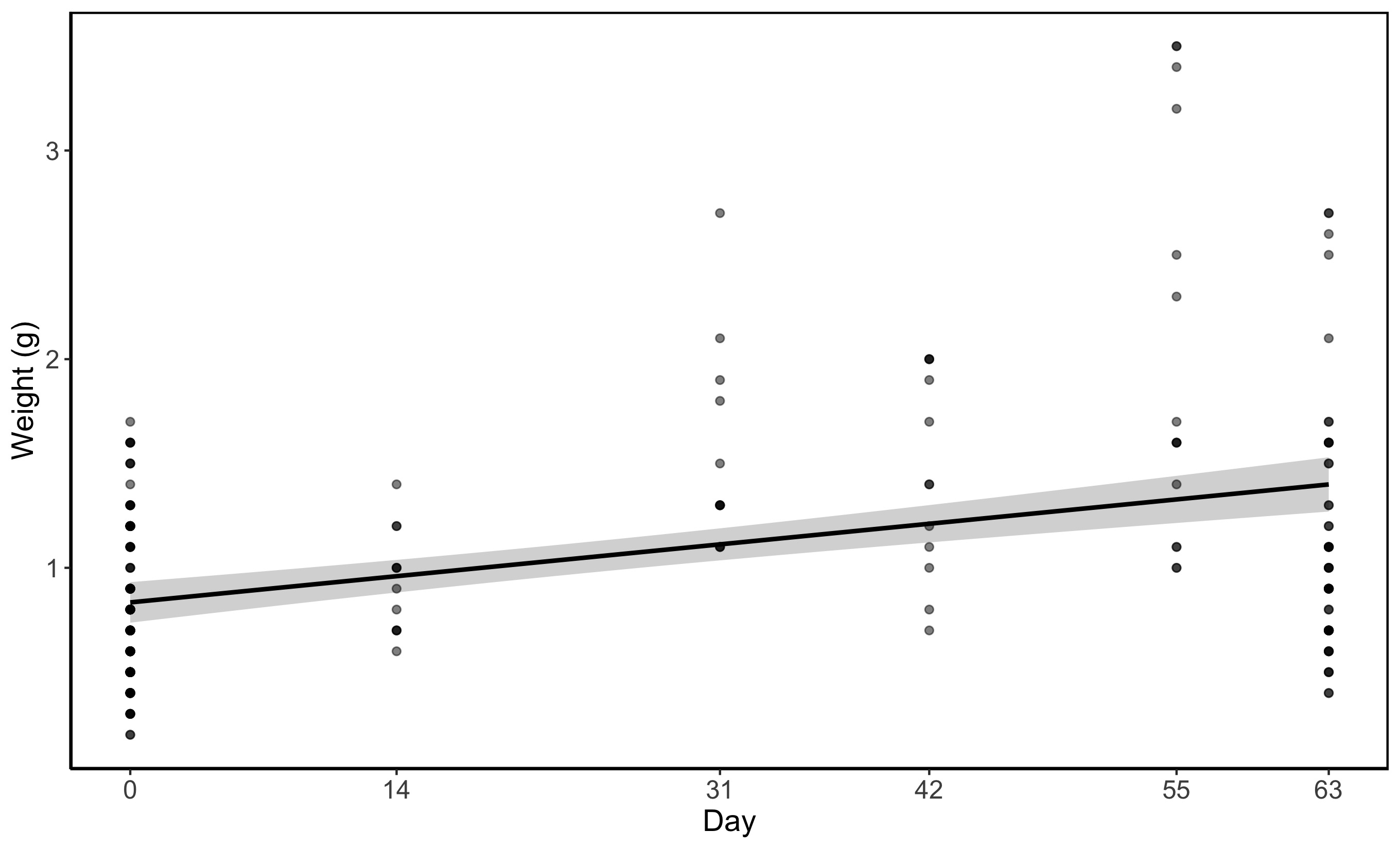

Supplement: S1 Fig — (JPG) [file pone.0284933.s001.jpg]

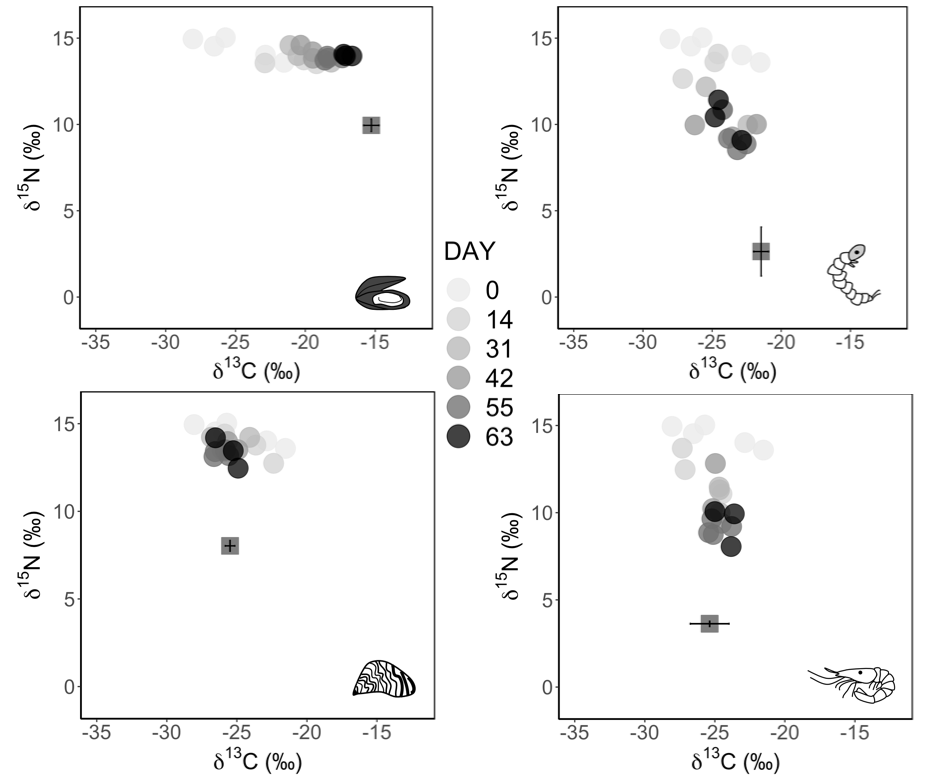

Supplement: S2 Fig — δ13C and δ15N of all feeding groups (A = Chilean mussels, B = chironomids, C = dreissenids, D = krill), with samples color coded by day of muscle sample collection. Squares indicate average isotope value for corresponding food items, while circles indicate round goby. (PNG) [file pone.0284933.s002.png]

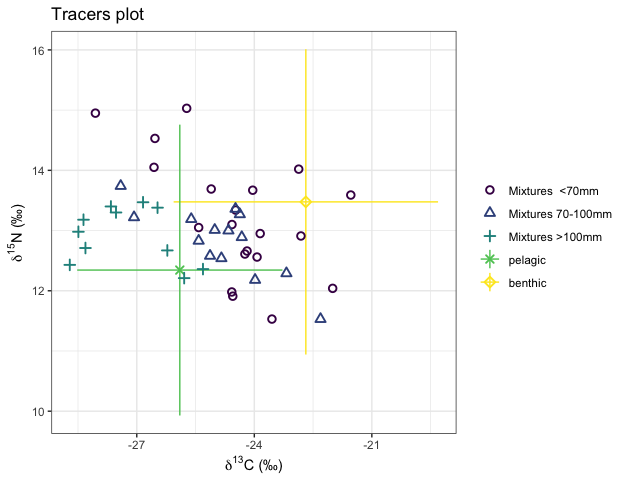

Supplement: S3 Fig — (TIFF) [file pone.0284933.s003.tiff]

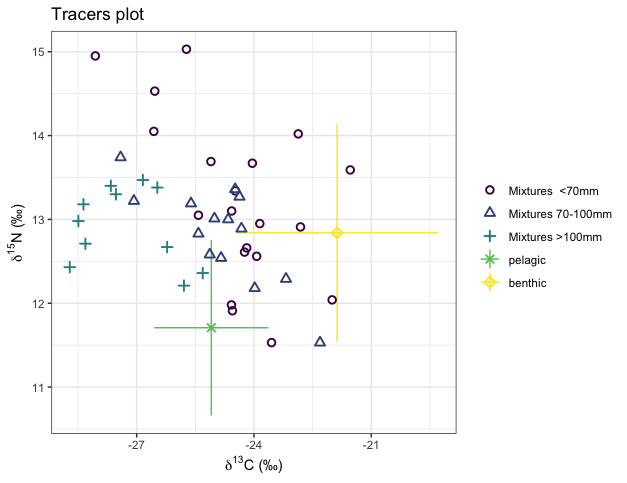

Supplement: S4 Fig — (TIFF) [file pone.0284933.s004.tiff]
