# Supplementary material for: Round goby (Neogobius melanostomus) δ13C/δ15N discrimination values and comparisons of diets from gut content and stable isotopes in Oneida Lake
Source: PLoS One. 2023 Apr 24;18(4):e0284933. doi: 10.1371/journal.pone.0284933 (PMC10124857; doi:10.1371/journal.pone.0284933)
Supplement: Attachment — Submitted filename: PLOS Response to Reviewers 2.docx [file pone.0284933.s007.docx]

Response to Reviewers

We thank the editor for the opportunity to edit and resubmit the manuscript. We carefully considered and attempted to address each of the reviewers’ comments.

Line numbers reflect those found in the revised manuscript.

**Reviewer #1**

Line 41: I don’t think that an emphasis on ‘sport fish’ is necessary; importance of effects shouldn't be determined by fish we 'care' about because we can fish for them. Changed “popular sport” to “native” fish in line 41.

Line 55-56: This critique is true if you only have one study at one time point, but becomes less of an argument if you have multiple studies (which you reference) that have collected diet at different time points. Included “without repeated sampling over time and space” in lines 56-57.

Line 58: ‘average’ is a better descriptor than ‘overall’. GCA works better for rare items; SIA captures the contribution of either the dominant food or averages the effects of many foods. Accepted following the reviewer’s suggestion.

Line 61-2: Here it would be good to actually discuss the reasons why discrimination factors differ, as they are the reason you argue you need specific factors for gobies. Included reasoning in lines 63-64.

Line 76: This is a prediction, not a hypothesis. Changed “hypothesized” to “predicted” in line 78.

Line 124: I believe ‘or’ instead of ‘to’ is more accurate word choice. Accepted following the reviewer’s suggestion.

Line 234: Please use a consistent number of significant digits throughout (eg., these are different from the abstract) Accepted following the reviewer’s suggestion.

Line 234 and 241: In the first line, you present the C discrimination factor as negative, but in the second line you did not. Please be consistent and in line with the literature. Our experimentally determined discrimination value is negative, while the standard value used in the literature is positive.

Table 2: Why does Δδ15N have a tau but Δδ13C does not? Removed the tau from 15N.

Line 302: Add ‰ to ‘-3’. Accepted following the reviewer’s suggestion.

Line 330: Please review the ms to make sure you are referring to δ and not absolute levels of C and N (i.e. make sure to use the delta notation when you are referring to changes in isotope ratios, as you are here, and not changes in the amount of carbon or nitrogen, which you are not measuring). Added delta notation where it was missing in the manuscript.

Line 344-5: Saying ‘shifting between the two sources’ suggests as they get larger, some are now specializing on benthic prey, which I believe is not what you intended you say. Deleted this sentence, line 408.

Line 345-6: But didn’t you sample gobies across several months? Are you suggesting that period just happens to be when they are all switching between a mixed diet to a pelagic (mussel) diet? I’m not really sure what you’re arguing in the latter half of this paragraph (which trend you’re trying to explain); is it ontogenetic diet shifts? Variability? Deleted this sentence, line 347.

Line 349: Specify these are deltaC values. Accepted following the reviewer’s suggestion.

Line 350: Again, please make sure you’re always providing the appropriate units and specifying what the numbers are. Accepted following the reviewer’s suggestion.

Line 349-353: Unclear comparison – if your value for amphipods is -22.3, why are you comparing that to perch values? Deleted this clause in line 353.

Paragraph line 347: Perhaps give more of a context for this paragraph – why is temporal and spatial variability in isotope values important? We have clarified the sampling schemes from Bowman and our approach are different. As a result, we should not expect stable isotope values to be identical, much less in a lake undergoing food web shifts, but are generally pleased with the correspondence between the two approaches (lines 353-354).

Line 362-3: So when you said earlier you measured shell fragments, does that mean the shells were intact in these smaller gobies? Wouldn’t that be important information to give from the GCA? It’s also unclear why these small gobies were consume items that they were not getting nutrition from – isn’t that a low-fitness strategy? Why would that behavioural trait persist? Good point, we think that sentence is too speculative and deleted it line 361.

Paragraph line 370: This paragraph is unclear. Round gobies invaded after zebra and quagga mussels? Native fish weren't declining before gobies were introduced? Are both quagga and zebra mussels declining in the lake? Are you implying the round gobies are responsible for the decline in quagga mussels? You last sentence is also just a thought, it seems, with no support; sure that's possible, but it's also possible they'll do fine together (how do gobies coexist with species in their native range?) Changes made to this paragraph (lines 368-376) to increase clarity, last sentence was deleted.

**Reviewer #3:**

Line 19. Remove “over the 63 days”, you already mentioned in line 17. Accepted following the reviewer’s suggestion.

Line 49. Include the reference [39] Accepted following the reviewer’s suggestion.

Line 52. Include the information of the reference 39. If is the same that this works reports previously. Accepted following the reviewer’s suggestion, now reference #13.

Line 63. Change, Post from 2002 by Post (2002) [14]. Accepted following the reviewer’s suggestion.

Line 83. Include, 114 round gobies “from” 26-54 mm in length… Accepted following the reviewer’s suggestion.

Line 114. Reference. Accepted following the reviewer’s suggestion.

Lines 117-119. How the fact that some prey items that are different from the area where the gobies were collected, allowing a better estimate of turnover rates and discrimination factor.
How can you deal with the fact that you have some item preys that were from Oneida Lake and some outside this area? We chose food sources originating from both Oneida Lake and outside the area intentionally for the feeding experiment to maximize the contrast between fish and prey. We expected sources from outside of the study area to be isotopically different from available food sources in the lake and provide an opportunity to easily measure discrimination rates. If the values were close together the effect would be smaller but the noise would be higher, so the ratio would decline. We also included foods that were similar to the types that would be found in the lake to ensure that the way they were processed in the goby would be similar. Included a clause in lines 121-122 to further elaborate this point.

Line 132. How much sample weight for SIA? Added (0.2-1.0 mg) to line 135.

Line 141. If you have values above 3.5, you need to normalize the data, as that is the set threshold. You mention that the range goes up to 3.72, those values need standardization. We followed this suggestion and normalized all fish with C:N values >3.5 following equation 2. This resulted in some changes in the initial delta C values which affected the discrimination factor. The experimental d13C discrimination value was recalculated, and all mixing models were re-run with the lipid-corrected data. Overall, this resulted in only minor changes to the model estimates.

Lines 189-190. Even though you include a “justification” for only used %FO, this justification should be with a reference that support the statement, if not, it is just an opinion, not a justification. Included a reference in line 193 (#23).

Line 190. What do you mean with “Round gobies are gape limited; therefore” it is talk about gape mouth? Or what gape? Yes, the size of prey items round gobies can consume is constrained by their mouth-size, in other words they are “gape limited.”

Line 209. Instead of used “the “standard” discrimination factors” … used the Post (2002) discrimination factors… Accepted following the reviewer’s suggestion.

Line 238. You mentioned before that the most informative prey item was Chilean mussels (Line 236-238). But then in line 238 said: “The next most informative were the chironomids“ which one is the correct? Changed “next” to “second” in line 241.

Table 3. You mentioned in lines 204 – 207: Amphipods and chironomids were considered one end member (benthic) because they generally feed on detrital food sources. Dreissenids were considered one end member because they feed directly on pelagic phytoplankton [14]. Amphipods, chironomids, and dreissenids were collected from Oneida Lake.
But when the table 3 appears, you only have 3 (carbon) and 4 (nitrogen) individuals for each, benthic and dreissenids groups. So, for benthic I want to know how many amphipods and chrinomids you have in the group, and it is a really a concern that the SD in the d13C values for benthic group is higher than 2%... it is a really really high variation… so, I don’t know if is due to the sample size, or the fact that you mix to different taxa or both. The benthic group included two chironomids and one amphipod collected from the field. One blood worm had a slightly more depleted lipid-corrected d13C value compared to the other two values from the amphipod and other blood worm. This, in combination with the low sample size, resulted in the high variation for d13C. We included a discussion about how our values were similar to those found by Bowman in Oneida lake in 2003, to try to address this concern. (line 348-356)

Lines 307-308. What do you mean with “credibility intervals”? Credible intervals are the Bayesian version of a confidence interval. The mixing models run in this paper produced 95% credible intervals.
Additionally, …“due to the two prey endmember groups having have similar isotopic values” … this is not true. With a difference higher than 2% do you think that the groups have the same values? For this statement be true you should have done a statistical analysis, otherwise it is just an opinion with no mathematical support. A t-test indicates the values are not statistically different, because of the high SD for benthic 13C value. Additionally, in the supplemental figures 3 &4 the values show significant overlap of their standard error bars, now referenced in line 312.

Lines 361-363. You started the phrase with “The smallest round goby observed to have consumed a dreissenid was 34 mm in length”, but then you mentioned that the “Round gobies smaller than 50 mm lack the pharyngeal teeth strength required to break open dreissenid shells” … so how do you explain that small individuals (34 mm), could feed on those mussels? We have included a statement on how small round gobies swallow can small dreissenids whole, but are likely not acquiring many nutrients (lines 360-363).
Ok, but if this food item does not provide a large amount of nutrients, and also cannot be opened to extract the individual... what is the explanation for that small gobies feeding of it?

As this was also a concern of reviewer 1, we removed this speculative sentence on line 361.

Mixing models and isotopic niche. What do you mean with pelagic and benthic in both isotopic niche graphics? It is better if you include all the food items A=Chilean mussels, B=chironomids, C=dreissenids, D=krill), and all the round goby isotopic information. Otherwise, pelagic and benthic, it doesn't tell me anything, there is no reference to what that is this. If you combine all the food items and the goby you could have a better idea about the behavior of the values, with this I mean, how far or close are the food items (prey) with respect of the goby (predators). Descriptions of what constitutes pelagic vs benthic food sources are in lines 207-210 and lines 274-276. The benthic endmember value was calculated using SIA values from the benthic macroinvertebrates chironomids and amphipods, while the pelagic endmember value was calculated as an average of dreissenid SIA values. The food items used in the feeding experiment cannot be used in the mixing models, because the mixing models were run on SIA data from wild caught fish, while the food items in the feeding experiment (with the exception of the dreissenids) are not representative of Oneida lake SIA prey values that wild fish would be incorporating, and were used exclusively as replicates to determine accurate, round goby-specific discrimination values.

Supplementary material S3 and S4.
If you create the better graphs you should mentioned the figures in the text. Figures are now mentioned in line 312.
